# Treatment of Fractures of the Patella in the Hospital of Prof. Langenbeck

**Published:** 1872-07

**Authors:** A. W. Calhoun

**Affiliations:** Vienna, Austria


### FOREIGN CORRESPONDENCE.

---

### TREATMENT OF FRACTURES OF THE PATELLA, IN THE HOSPITAL OF PROF. LANGENBECK.

BY A. W. CALHOUN, M.D.

Various have been the efforts and means adopted in the treatment of this injury with the view of bringing about bony union between the fragments, and the experience of most surgeons tells us that the accomplishment of this object is by no means an easy matter, since so many obstacles interfere to retard and prevent the so much wished-for result. In comparison with such injuries to other bones of the body, this fracture occurs extremely seldom; yet the difficulty of obtaining firm connection between the parts, and the evil consequences resulting to the individual from such a failure, would lead us to seek in all directions, and adopt all reasonable ideas calculated to assist in so desirable a termination as complete or bony union.

In a few cases, the fracture is oblique, but the greater number are transverse—so produced by a combination of causes, such as a blow or fall directly upon the patella itself, or the sudden and violent contraction of the muscles (quadriceps extenso) attached to its upper border, as occurred in a case to be mentioned. It is surprising that the patella is so comparatively seldom the seat of such a trouble—more especially when we take into consideration its constant exposure, and also when we remember that the clavicle and olecranon processes, firmer in texture, and about equally as much, but no more exposed, are almost continually receiving hurts that result in their different fractures. The patella, however, rests posteriorly upon cartilaginous substance, which takes off, to a great degree, the volume of a direct blow, depending upon the spongy and somewhat elastic qualities; and again, this bone is movable in all directions, and thus readily yields under any directly-applied force. These facts account, to some extent, for its few injuries when compared to those of the clavicle and olecranon process, which have neither the mobility nor, so to speak, the protecting media of the patella.

The causes of ill success, in the majority of attempts at a perfect cure, are most frequently due to a want of continuous apposition between the separated portions, or to the bulging of fluids and the formation of coagula between the fragments. Of course, with any of these accidents occurring, nothing but ligamentous connection will ensue; and the plan that will bring about the one (continuous apposition), and prevent the other (ligamentous union), is most apt to succeed to a happy termination, and should be the one adopted.

No doubt but that every surgeon, who has had much experience in the treatment of such affections, has some peculiar method followed by himself with more or less success—such, for instance, as the "iron ring treatment," suggested by Prof. Paul Eve, of Nashville, Tennessee, a few years ago, and which, on account of its simplicity in application and the success of reported cases, is well worthy of trial; and some may have practiced a similar treatment to that—the subject of this communication—which by no means is entirely new in all its points, but which I give as the plan of Prof. Langenbeck, used in his clinic and hospital, and followed now for quite a number of years, and in an exceedingly large number of cases, and with most gratifying results, viz: in a very large majority of instances (so far as could be positively judged), firm and bony union, and subsequent good use of the injured limb. Dr. Busch, first clinical assistant, was kind enough to furnish me with some statistics of cases thus treated under his care, and they show almost invariably favorable results.

I can, perhaps, more clearly and concisely describe the method by giving a case of recent fracture brought into the clinical room, and its treatment there, and its after-management.

Herr H. M——, aged thirty-five, while walking along the streets, stepped upon a potato peeling, which caused him to slip and fall forward. In attempting to recover himself, he threw his body backward with considerable force, when he "heard a snapping noise, and felt his left leg give way underneath him," and fell immediately to the ground. A sharp, cutting pain passed through the knee-joint, the violence of which ceased after a few moments. With some assistance, he raised himself in a standing position, but found that he was unable to walk, or even stand upon the left limb. Was taken home, and treated by some "*house remedies*," which failing to relieve him of his pain, or to strengthen his enfeebled limb, he was brought to the clinic on the following day (December 4th, 1871), when, upon examination, the patella was found fractured transversely, and the upper fragment drawn upward by strong contractions of the quadriceps extensor muscle, leaving an intervening space of nearly one inch between the two separated ends. A light inflammation immediately over the patella, with a moderate amount of swelling, and inability to extend the leg when slightly flexed, were the only externally apparent symptoms of any injury.

Having extended the leg thoroughly, and having brought the two fragments in close and exact apposition, by pressure with the fingers, placed several turns of broad adhesive strips around the limb, above and below the knee, and so arranged as to draw toward each other, and thus hold the parts firmly in position. Over this, and extending from foot to hip-joint, is laid the cloth bandage (flannel being usually preferred), which controls the action of the muscles, and also assists the adhesive strips. Two thin pieces of gutta-percha, three or four inches in width and ten or twelve in length, softened in hot water, and thus made pliable, are laid over this—the one above, and the other below, and pressing and supporting the upper and lower edges of the patella. It is best, when the pieces are also so placed, that the opposing ends on the sides of the knee look somewhat toward each other, as in this position they better resist the subsequent strain brought to bear upon them. After a few turns more of the roller over these strips, for the purpose of ficiently holding them in their places, a "Malgaigne's Clamp" is applied, with the two sets of hooks fastened above and below in the gutta-percha, and the connecting screw turned to the extent of bringing the hooks with the strips nearer each other, and consequently forcing the fractured parts still more firmly in apposition. The entire limb is next enveloped in a plaster of Paris bandage, and the patient placed in bed.

Where much swelling or effusion of blood around the knee follows the hurt, this last bandage must, of course, be several times renewed, till the parts have regained their normal size, as upon the reduction of the swelling and the absorption of the effusion, the bandage becomes too loose to afford any support.

In this case, no other treatment whatever was followed, and at the end of the fifth or sixth week, the patient was walking about the hospital grounds, with no further protection around his knee, save the common roller bandage, the fracture having securely and strongly united during these few weeks of absolute rest.

In the first use of these "Clamps," the hooks were barbarously fastened in the flesh itself; but, aside from the intense inflammation and pain thus produced, no good result was reached, since, through suppuration and tearing of the parts, the hooks would fall out of place of their own accord, after a few days, leaving again the fragments of the patella widely apart. Then they were imbedded in the bandage, and afterward in the plaster of Paris; but upon tightening the screws of the instrument, the bandages also gave way under the strain. Finally, the gutta-percha strips, softened and suitably fitted to the limb, were found, after cooling, sufficient to hold the instruments, and withstand any amount of force brought to bear upon them. As a rule, the patients are to be kept in bed from three to six weeks, according to circumstances; yet Prof. Langenbeck reports cases in which perfect cures with bony union ensued in fourteen to twenty-one days.

Prof. Langenbeck claims for this mode of treatment greater influence upon the parts directly in question, and less trouble or inconvenience both to surgeon and patient, and that a greater per cent. of good results follow. Although this plan may have been, in part, often before practiced, yet, as a whole, the method is so simple and the statistics so favorable as to recommend it to all who may have to do with such fractures.

VIENNA, AUSTRIA, July 22, 1872.

